# A Quantization-Based Multibit Data Fusion Scheme for Cooperative Spectrum Sensing in Cognitive Radio Networks

**DOI:** 10.3390/s18020473

**Published:** 2018-02-06

**Authors:** Yuanhua Fu, Fan Yang, Zhiming He

**Affiliations:** 1 School of Information and Communication Engineering, University of Electronic Science and Technology of China, Chengdu 611731, China; 201411020323@std.uestc.edu.cn (F.Y.); zmhe@uestc.edu.cn (Z.H.); 2 Institute of Electronic and Information Engineering of University of Electronic Science and Technology of China in Guangdong, Dongguan 523808, China

**Keywords:** cognitive radio, multibit cooperative spectrum sensing, quantization, hard fusion, soft fusion

## Abstract

Spectrum sensing remains a challenge in the context of cognitive radio networks (CRNs). Compared with traditional single-user sensing, cooperative spectrum sensing (CSS) exploits multiuser diversity to overcome channel fading, shadowing, and hidden terminal problems, which can effectively enhance the sensing performance and protect licensed users from harmful interference. However, for a large number of sensing nodes that need high bandwidth of the control channel for data transmitting, CSS increases cooperative overhead. To address this problem, we investigated the soft decision fusion strategy under a limited bandwidth of the control channel and proposed a simple quantization-based multibit data soft fusion rule for CSS for its simple structure and easily implementation. Under the quantization-based sensing strategy, each cooperative secondary user (SU) adopts an energy detector for local spectrum sensing. Each SU transmits quantized multibit data that sends local sensing information, instead of forwarding local one-bit hard decision results or original observation statistics, to the fusion center (FC). Furthermore, the closed-form expressions of the quantization levels and the quantization thresholds are analytically derived. Simulation results indicate that the detection performance of the proposed method approaches that of the conventional soft fusion rule with less cooperative overhead and outperforms the hard decision rules. Extensive simulations also show that multibit quantization fusion achieves a desirable tradeoff between the sensing performance and the control channel overhead for CSS.

## 1. Introduction

Cognitive radio is a promising technology to solve the problem of spectrum resource scarcity and improves the efficiency of spectrum utilization. In cognitive radio networks (CRNs), secondary users (SUs) can opportunistically access the specific spectrum channel that is assigned to primary users (PUs) for data transmission [1]. This process requires accurate and reliable spectrum sensing at SUs before the SUs start to communicate. More precisely, spectrum sensing technology plays a key role in identifying which portion of the licensed channels is available for SUs. Therefore, spectrum sensing has been attracting significant interest in the context of CRNs in recent years.

In the spectrum sensing process, various transmission impairments of the sensing channels (i.e., the wireless channels between the PUs and the SUs), such as severe fading, shadowing, and hidden terminal problems, have a crucial impact on the system’s detection performance [2]. Thus, the detection performance of local single-user sensing is limited and may not be reliable. In order to enhance the reliability and mitigate these effects, recent research progress shows that cooperative spectrum sensing (CSS) is a promising technique that can combat the effect of transmission impairments [3,4,5]. In a CSS system, local SUs report their sensing information to the fusion center (FC) in which a global test statistic is constructed for a final decision. From a resource optimization perspective, joint design PHY-layer spectrum sensing and MAC-layer resource scheduling was presented in [6]. Under this framework, the sensing confidence level was introduced to characterize the presence of imperfect sensing. In [7], the resource allocation scheme for cognitive femtocell users was investigated. To further improve spectrum holes utilization efficiency [8], a high-order hidden bivariate Markov model-based spectrum hole prediction method was proposed in [9]. Under CSS schemes, data fusion and final decision making can be executed in two modes, namely, centralized CSS or decentralized CSS [10]. In the centralized mode, there is an FC to collect sensing information from each local SU and makes a final decision about whether the spectrum channel is available for SUs. On the other hand, in decentralized mode, since the FC does not exist, SUs exchange their sensing information with other neighboring SUs and the final decision is made by any one of the SUs. Due to the limited communication resource between SUs and the FC, throughout this paper, we focus on the problem of CSS with multibit quantized data fusion in the centralized mode.

There are two types of sensing results in the centralized CSS. (I) Soft decision: Each local sensing node reports its original observed test statistics to the FC and then combines all the received observations to determine whether the PU signal is present or absent. This scheme may require more communication resources and energy consumption for a large number of sensing nodes. (II) Hard decision: Each cooperative SU compares the test statistics with a predefined threshold and then generates a one-bit binary decision data; for instance, Bit 1 represents the existence of a PU signal, while Bit 0 represents the absence of a PU signal [11]. Each local one-bit decision result is reported to the FC, and a final decision is made by specific fusion rules such as OR-logic, AND-logic, and MAJORITY-logic [12]. Even though soft combining outperforms hard combining, sending whole test statistics increases the bandwidth requirement of the control channel, which may be infeasible in practice. The hard fusion rules require less reporting overhead at the cost of degrading the sensing performance. Hence, in order to reduce the control channel overhead and achieve reliable sensing performance simultaneously, each SU should quantize their local observations into multiple decision regions [13,14].

Reporting quantized local log-likelihood ratio (LLR) decision values to the FC can enhance CSS performance with less cooperative overhead [15,16]. In [16], an optimal quantizer using a Lloyd–Max algorithm based on the local node’s LLR value was presented. However, this scheme requires prior knowledge of the PU signal, which may not available in practice. In [17], from the throughput maximum perspective, a multibit quantization scheme was introduced. Under this scheme, we require the prior probability that a PU is active or passive to determine the quantization interval. A scheme of cluster-based CSS with adaptive thresholds and multi-bit local decision was presented in [18]; in this paper, a cluster decision was first made and then produced a global decision, but the method on how to determine the cluster was not given. A softened two-bit fusion rule was proposed in [19,20] to decrease the reporting channel overhead. According to this scheme, the complete observed test statistics at each SU is mapped to four distinct portions, which are separated by two thresholds. If the test statistics fall into one of these portions, then two bits of data is assigned to it. In [21], a linear combining quantized data using uniform quantization was investigated. The number of quantization bits and combining weight were jointly optimized to maximize the detection probability. In [22], a tradeoff was investigated to illustrate the relationship between the reported quantization bits and system throughput of CSS. The optimal parameters such as the number of quantization bits and the global decision threshold were determined by maximizing the normalized throughput under a certain probability of detection constraint. A quantization strategy adopting energy detector based on soft fusion rule of CSS was investigated in [23]. The analytical expression of the quantization criterion and the associated probability of a false alarm were presented. Although these papers mentioned above investigate quantized soft data combing, they are still not tractable enough to be used as a cost function for parameter optimization. The computational complexity of such a scheme is high. The closed-form expressions of the quantization thresholds are not given. Therefore, an efficient and simple quantization-based multibit data fusion scheme is indispensable for CSS in CRNs.

In this paper, a simple quantization-based multibit data soft fusion rule for CSS is presented to achieve a desirable tradeoff between sensing performance and control channel overhead. Under this scheme, each local SU adopts an energy detector to estimate the energy of received signal during a sensing interval. This energy value is compared with a pair of quantization thresholds and then produces multibit data. The FC collects quantized multibit data from all SUs and performs inverse quantization based on the received data of each SU. A global test statistic, namely, the sum of the inverse quantization values, is constructed at the FC to decide whether a PU signal is present or absent. The main contributions of this paper are concluded as follows.

(1) We propose a simple quantizer design scheme. From the global false alarm probability at the FC perspective, we first determine the center quantization thresholds at each secondary user according to Neyman–Pearson criteria. This scheme is different from the scheme proposed in [17]. Meantime, we establish the “3σ” rule to design the quantization interval, and closed-form expressions of the quantization levels and thresholds are derived. Compared to the suboptimal linear-quantization multibit combining (SLMC) scheme in [24], which employs a numerical search algorithm to find the optimal quantization interval, we provide a computationally affordable method to determine quantization parameters.
(2) We also analyze the detection performance of CSS based on the soft fusion rule (equal gain combining) and the hard decision rule, and investigate the tradeoff between control channel overhead and sensing performance under the proposed scheme. Furthermore, the effects of the number of cooperative SUs, the number of quantization bits, and the number of samples for local sensing on detection performance are examined. Simulation results may be useful to determine parameters to meet the required performance in a practical CSS system.
(3) Lastly, extensive numerical simulation experiments are performed to demonstrate the proposed quantization fusion rule and compare it with soft fusion, hard decision, the SLMC [24] scheme, and semi-soft [12] fusion rules. Overall, the proposed quantization scheme achieves a desirable tradeoff between the sensing performance and the control channel overhead, with low computational complexity.

The rest of the paper is organized as follows. The system model and detection problem is formulated in Section 2. The proposed simple quantization-based multibit data fusion scheme as the associated procedure of the algorithm is given in Section 3. Numerical simulation results under different scenarios are presented in Section 4. Finally, the main conclusions are summarized in Section 5.

## 2. The System Model and Problem Formulation

**Figure 1:**
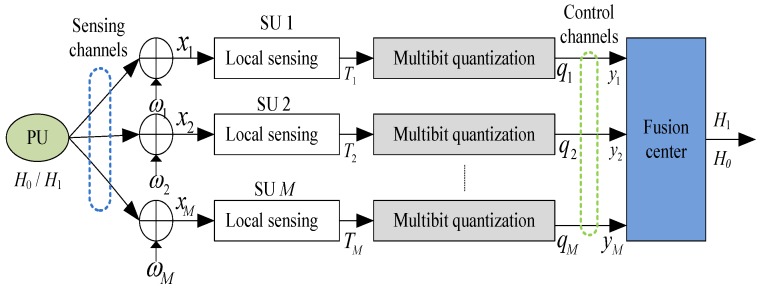
The structure of quantization-based cooperative spectrum sensing (CSS) system with *M* secondary users (SUs) and a fusion center (FC).

We consider a CSS system in CRNs, including a PU, *M* spatially distributed SUs, and an FC. Specifically, each SU performs local spectrum sensing. Due to the control channel bandwidth and energy supply limitations, the local sensing results from SUs have to be quantized before transmitting them to the FC. Assume that the control channel is error-free, this is reasonable by using an efficient channel coding mechanism to mitigate the noise and interference in the reporting phase. Let the null hypothesis (*H*_0_) and the alternative hypothesis (*H*_1_) denote that a PU signal is absent and present, respectively. Figure 1 shows the basic structure of a quantization-based CSS system.

According to the structure model given in Figure 1, the detection problem can be described as follows. Whether a PU signal is present or not is decided by combining *M* independent, individual local sensing nodes quantized data at the FC, and a global test statistic is constructed for making a final decision on the presence/absence of the PU signal by a certain fusion rule under a global false alarm probability constraint.

### 2.1. Local Spectrum Sensing

Spectrum sensing can be formulated as the following binary hypothesis testing problem [25]:

*H*_0_: *x_i_*(*n*) = *w_i_*(*n*) *H*_1_: *x_i_*(*n*) = *s*(*n*) + *w_i_*(*n*) (1)

where *x_i_*(*n*) denotes the *i-*th SU received signal sample at time instant *n*, *s*(*n*) is the primary signal with a received power of $\sigma_{s}^{2}$ (the effect of fading can be absorbed in $\sigma_{s}^{2}$), *w_i_*(*n*) represents the *i*-th SU additive white Gaussian noise (AWGN) at time instant *n* with zero mean and known variance $\sigma_{i}^{2}$. Furthermore, assume that the noise *w_i_*(*n*) and primary transmitted signal *s*(*n*) are independent of each other.

The energy detector is used for the local sensing due to its low complexity and needing no a priori knowledge about the PU signal. The *i*-th SU received energy *T_i_* over the *N* samples is given as

(2) $$T_{i}=\frac{1}{N}\sum_{n=1}^{N}{\left|x_{i}(n)\right|}^{2}$$

when *N* is large enough, using the central limit theorem (CLT), the test statistics *T_i_* follows an asymptotically Gaussian distribution with mean and variance as [26]

(3) $$T_{i}\sim \;\left\{ \begin{array}{ll} N(\sigma_{i}^{2},2\sigma_{i}^{4}/N) & H_{0}\\ N((1+\gamma_{i})\sigma_{i}^{2},2{\left(1+\gamma_{i}\right)}^{2}\sigma_{i}^{4}/N) & H_{1} \end{array}\right.$$

where N(μ,σ) is a normal distribution with mean *u* and variance σ, $\gamma_{i}$ is the SNR, and $\gamma_{i}=\sigma_{s}^{2}/\sigma_{i}^{2}$, $\sigma_{s}^{2}$ is the variance of the PU signal.

### 2.2. Local Hard Decision Fusion

In the hard decision fusion, each SU after a specific sensing interval then makes a binary decision independently about the presence or absence of a PU signal. Each *i*-th SU sends its one-bit decision *d_i_* to the FC via a common control channel, where

(4) $$d_{i}=\left\{ \begin{array}{ll} 1 & H_{1}\\ 0 & H_{0} \end{array}\right.$$

The FC combines local decision results from all cooperative SUs by a certain fusion rule for making a final decision. The general form of the hard fusion rule can be expressed as

(5) $$\sum_{i=1}^{M}d_{i}\overset{H_{1}}{\underset{H_{0}}{≷}}K$$

In Equation (5), the fusion rule is OR-logic when *K* = 1, AND-logic when *K* = *M*, and MAJORITY-logic when K>⌈M/2⌉. The global probability of detection *Q_d_* and global probability of false alarm *Q_f_* for AND, OR, and *K*-out-of-*M* (*KM*) rules are given as follows:

(6) $$Q_{dAND}=\prod_{i=1}^{M}P_{di}$$

(7) $$Q_{fAND}=\prod_{i=1}^{M}P_{fi}$$

(8) $$Q_{dOR}=1-\prod_{i=1}^{M}\left(1-P_{di}\right)$$

(9) $$Q_{fOR}=1-\prod_{i=1}^{M}\left(1-P_{fi}\right)$$

(10) $$Q_{dKM}=\sum_{i=K}^{M}\left({}_{i}^{M}\right)P_{di}^{i}{\left(1-P_{di}\right)}^{M-i}$$

(11) $$Q_{fKM}=\sum_{i=K}^{M}\left({}_{i}^{M}\right)P_{fi}^{i}{\left(1-P_{fi}\right)}^{M-i}.$$

### 2.3. Soft Decision Fusion

In soft decision fusion, every cooperative sensing node sends its complete test statistics to the FC. The statistics from all cooperative nodes are then constructed via a global test statistic by the soft fusion rule. The soft fusion rule can be adopted as equal gain combining (EGC), maximal ratio combining (MRC), and the optimal likelihood ratio test (LRT) [27]. In this paper, FC compares the sum of all cooperative nodes test statistics with a predefined decision threshold. The global probability of detection *Q_d_* and the probability of false alarm *Q_f_* using the EGC fusion rule at the FC is given as

(12) $$Q_{d}=Q(\frac{\lambda_{c}-\sum_{i=1}^{M}(1+\gamma_{i})\sigma_{i}^{2}}{\sqrt{\frac{2}{N}\sum_{i=1}^{M}(1+\gamma_{i})\sigma_{i}^{4})}})$$

(13) $$Q_{f}=Q(\frac{\lambda_{c}-\sum_{i=1}^{M}\sigma_{i}^{2}}{\sqrt{\frac{2}{N}\sum_{i=1}^{M}\sigma_{i}^{4}}})$$

where *M* is the number of cooperative nodes, $\lambda_{c}$ is the predefined decision threshold in FC, *Q*(*x*) is the *Q*-function, and *Q*(*x*) is given as

(14) Q(x)=12π∫x+∞e−t2/2dt

**Figure 2:**
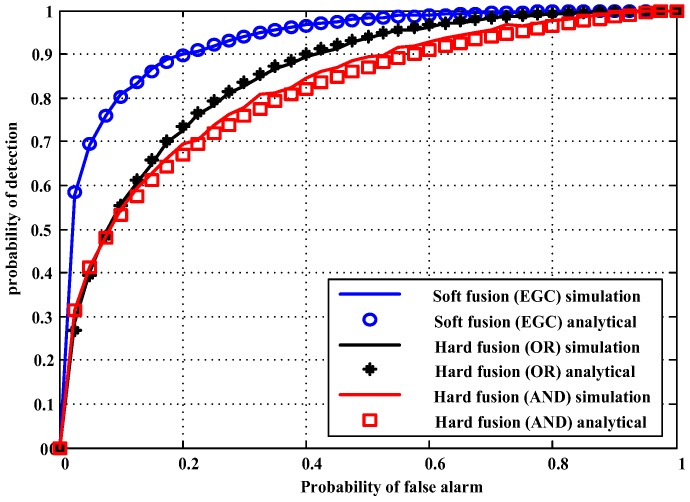
Probability of detection versus probability of false alarm of soft and hard fusion rules.

Figure 2 shows the global probability of detection versus the global probability of false alarm of the hard OR and AND fusion rules and the soft (EGC) fusion rule. The detection performances of these rules were evaluated. Ten thousand independent Monte Carlo simulation trials verified the theoretical results. Assume that all nodes have an SNR of −12 dB, the number of cooperative SUs *M* is 6, and the samples *N* is 400. We observed that the soft fusion rule achieved a higher detection probability than either of the hard fusion rules, and that the detection probability of the hard OR rule outperformed AND rule. Meanwhile, the theoretical results are well matched with the simulation results.

## 3. Proposed Multibit Quantization in Each SU

### 3.1. q-Bit Quantizer

In this work, the computed local test statistics *T_i_* has to be quantized before it is sent to the FC. The FC combines these quantized versions of *T_i_* using the EGC fusion rule to make the final decision. Generally, each SU adopts a *q*-bit quantizer, the test statistics *T_i_* is compared with a set of quantization thresholds ${\left\{\lambda_{i,k}\right\}}_{k=0}^{2^{q}}$ with $\lambda_{i,0}=0$, $\lambda_{i,2^{q}}=+\infty$. Let ${\left\{E_{i,k}\right\}}_{k=1}^{2^{q}}$ and $\Delta_{i}$ denote the quantization levels and quantization interval at each SU. The quantization rule is given as

(15) $$q_{i}=\psi_{i}(T_{i})=d_{i},\;if\;\lambda_{i,k-1}\leq T_{i}<\lambda_{i,k}\;\;i=1,2,\ldots ,M,k=1,\ldots ,2^{q}$$

**Figure 3:**
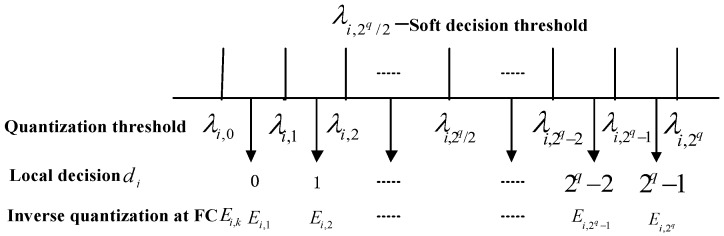
Proposed *q*-bit quantizer.

In Equation (15), $\psi_{i}(•)$ represents the quantization process at SU *i,* and $d_{i}\in {\left\{0,1\right\}}^{q}$ denotes a binary codeword. $\lambda_{i,k-1}$ and $\lambda_{i,k}$ denote the (*k* − 1)-th and *k*-th quantization boundaries at SU *i*, respectively. Figure 3 shows the proposed *q*-bit quantizer.

### 3.2. Multibit Combining and Decision Making

Under the multibit cooperative detection scheme, the quantization interval and thresholds should be determined firstly. Since the probability density function (PDF) of the energy test statistics *T**_i_*** is available for each sensing node, we designed a “3σ” rule to determine the quantization interval $\Delta_{i}$, which can be calculated as

(16) $$Z_{i,1}=\left\{ \begin{array}{ll} 0 & if\;S_{i,0}\leq 0,S_{i,1}\leq 0\\ \min(S_{i,0},S_{i,1}) & \;else \end{array}\right.$$

(17) $$Z_{i,N}=X_{i}$$

(18) $$\Delta_{i}=\frac{Z_{i,N}-Z_{i,1}}{2^{q}}$$

where

(19) $$S_{i,0}=\sigma_{i}^{2}-3\times \sqrt{2\sigma_{i}^{4}/N}$$

(20) $$S_{i,1}=\sigma_{i}^{2}(1+\gamma_{i})-3\times \sqrt{2{\left(1+\gamma_{i}\right)}^{2}\sigma_{i}^{4}/N}$$

(21) $$X_{i}=\sigma_{i}^{2}(1+\gamma_{i})+3\times \sqrt{2{\left(1+\gamma_{i}\right)}^{2}\sigma_{i}^{4}/N}$$

After $\Delta_{i}$ is determined, the quantization energy levels ${\left\{E_{i,k}\right\}}_{k=1}^{2^{q}}$ and thresholds ${\left\{\lambda_{i,k}\right\}}_{k=0}^{2^{q}}$ can be given by

(22) $$E_{i,k}=\left\{ \begin{array}{ll} \lambda_{i,\frac{2^{q}}{2}}-(\frac{2^{q}}{2}-k+\frac{1}{2})\Delta_{i} & 1\leq k\leq \frac{2^{q}}{2}\\ \lambda_{i,\frac{2^{q}}{2}}+(k-\frac{2^{q}}{2}-1+\frac{1}{2})\Delta_{i} & \frac{2^{q}}{2}<k\leq 2^{q} \end{array}\right.$$

(23) $$\lambda_{i,k}=\left\{ \begin{array}{ll} \lambda_{i,\frac{2^{q}}{2}}-(\frac{2^{q}}{2}-k)\Delta_{i} & 1\leq k<\frac{2^{q}}{2}\\ \lambda_{i,\frac{2^{q}}{2}}+(k-\frac{2^{q}}{2})\Delta_{i} & \frac{2^{q}}{2}<k\leq 2^{q}-1 \end{array}\right.$$

In Equations (22) and (23), $\lambda_{i,2^{q}/2}$ is the center threshold of quantization as $\lambda_{i,2^{q}/2}=\lambda_{c}/M$, the calculation of $\lambda_{c}$ is based on Equation (13) according to Neyman–Pearson criterion for a given $Q_{f}=\alpha$.

The FC collects all cooperative SUs quantized data, and the global test statistic $T_{c}$ is constructed as the sum of the received quantized data. That is,

(24) $$T_{c}=\sum_{i=1}^{M}\psi_{i}^{-1}(q_{i})$$

where $\psi_{i}^{-1}(•)$ represents the inverse of the quantization process at SU *i*, $\psi_{i}^{-1}(q_{i})$ represents the quantization levels ${\left\{E_{i,k}\right\}}_{k=1}^{2^{q}}$ at SU *i*, which is determined by Equation (22), and *T_c_* is compared with a predefined global decision threshold, $\lambda_{c}$, which is determined according to Equation (13) to decide whether a PU is present or absent, i.e.,

(25) $$T_{c}\overset{H_{1}}{\underset{H_{0}}{≷}}\lambda_{c}$$

The detection performance of the CSS with multibit quantized data is measured by the global detection probability $Q_{d}=\mathrm{Pr}(T_{c}\geq \lambda_{c}|H_{1})$ and the global false alarm probability $Q_{f}=\mathrm{Pr}(T_{c}\geq \lambda_{c}|H_{0})$.

The procedure of the proposed quantization-based multibit data soft fusion scheme consists of three main steps: a sensing request, energy detection, and decision making. When FC broadcasts a spectrum sensing request, the FC obtains the estimation of noise power $\sigma_{i}^{2}$ of each SU, then computes the center quantization threshold and reports it to each SU. After all SUs receive the threshold, each SU performs energy detection and produces multibit data that is transmitted to the FC through the control channel. The FC computes the sum of the inverse quantization vale for final decision making. The procedure of the proposed scheme is summarized in Algorithm 1.

**Algorithm 1. Proposed Quantization-Based Multibit Data Fusion Scheme****Sensing request:** 1: The FC broadcasts a spectrum sensing request, and each SU reports its noise power $\sigma_{i}^{2}$ to the FC for computing the center threshold of quantization $\lambda_{i,2^{q}/2}=\lambda_{c}/M$ according to (13). **Energy detection:** 2: The *i*-th SU computes its energy *T_i_* and quantizes data *q_i_* according to Equations (2) and (15), respectively. 3: Each SU reports its quantized data *q_i_* to the FC. **Decision making:** 4: The FC computes each SU inverse quantization value ${\left\{E_{i,k}\right\}}_{k=1}^{2^{q}}$ according to Equation (22) corresponding to the data obtained in Step 3. 5: The FC softly combines the data ${\left\{E_{i,k}\right\}}_{k=1}^{2^{q}}$ and constructs the global test statistic *T_c_* according to Equation (24). 6: Final decision making: if *T_c_*
≥
$\lambda_{c}$, the FC decides if a PU signal is present; otherwise, the FC decides that the PU signal is absent.

The computational complexity of our proposed algorithm is low. Since the quantization parameters are obtained based on closed-form analytical expressions, it is tractable enough to be used. For identically distributed SU observation statistics, the computational complexity increases linearly in 2*^q^* − 1. For non-identically distributed observation statistics, the complexity increases linearly in $M\times (2^{q}-1)$.

## 4. Simulation Results and Valuations

In this section, simulation results are presented to evaluate the performance of a quantization-based multibit data fusion CSS scheme. Without loss of generality, we assume that the PU signal *s*(*n*) is also a Gaussian random process with mean zero and variance $\sigma_{s}^{2}$ as the SNR, and noise variance $\sigma_{i}^{2}=\sigma^{2}=1$ for all cooperative SUs. We also consider, as references, the SLMC [24] scheme and the semi-soft fusion rule [12] for ideal control channel environment. The decision statistics of SLMC are given as

(26) $$T_{SLMC}=\sum_{i=1}^{M}u_{i}$$

In the SLMC fusion rule, $u_{i}\in \{0,1,\ldots ,2^{q}-1\}$, the decision threshold $\lambda_{u}$ at FC is selected in {0, 1, …, *M*(2*^q^* − 1)}, which is a positive integer.

### 4.1. Detection Performance

Firstly, we evaluate the detection performance of the proposed quantization-based soft fusion rule. We set parameters such that *M* = 6 total cooperative SUs, each SU received samples is *N* = 400 at a certain sensing interval, and the SNR is −12 dB for all SUs.

**Figure 4:**
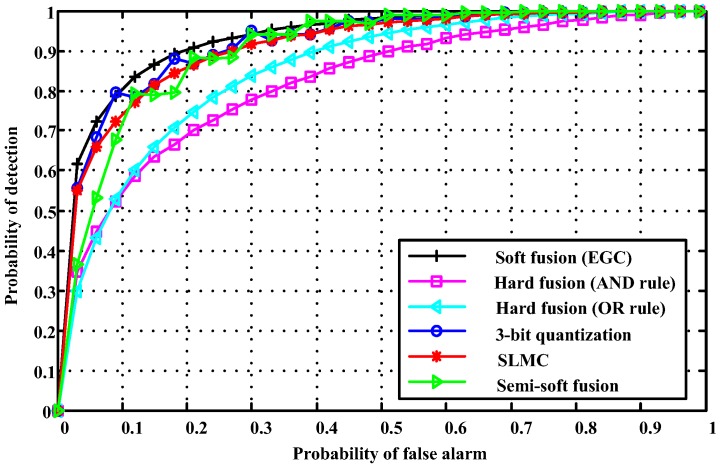
Receiver operating characteristics (ROC) curves of the proposed 3-bit quantization fusion scheme in comparison to the soft fusion (EGC), hard fusion (AND), hard fusion (OR), SLMC fusion, and semi-soft fusion schemes.

**Figure 5:**
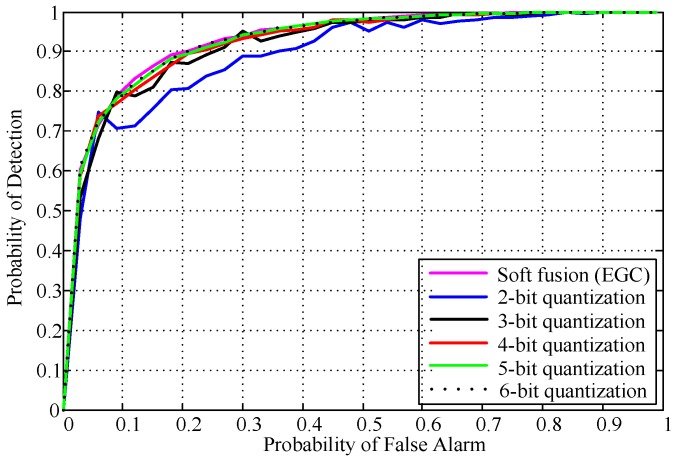
ROC curves of the proposed quantization-based detector for various quantization bits (*N* = 400, *M* = 6, SNR = −12 dB).

Figure 4 presents the ROC curves of the proposed multibit quantization detector with the hard OR and AND fusion rules, the soft fusion (EGC) rule, the SLMC fusion scheme, and the semi-soft fusion scheme. The detection probability of the 3-bit quantization-based soft fusion rule outperforms the SLMC fusion rule, the semi-soft fusion rule, and the hard fusion rules. Meanwhile, compared to the soft (EGC) fusion rule, the performance loss of the proposed scheme is negligible. To further evaluate the effect number of quantization bits on detection performance, the results in terms of the probability of detection for different quantization bits are given in Figure 5. We also found that the performance gain saturates when quantization bits are greater than 4. In particular, the detection performance of 4-, 5- or 6-bit quantization-based soft fusion rules is comparable to the soft fusion (EGC) rule without quantization. However, in practice, the number of quantization bits selected depends on the tradeoff between the available resource and the system’s required performance. For instance, the 2-bit and 3-bit quantization detector achieves a probability of detection of about 0.7 and 0.8 with less cooperative overhead, respectively, when the probability of false alarm is fixed at 0.1.

**Figure 6:**
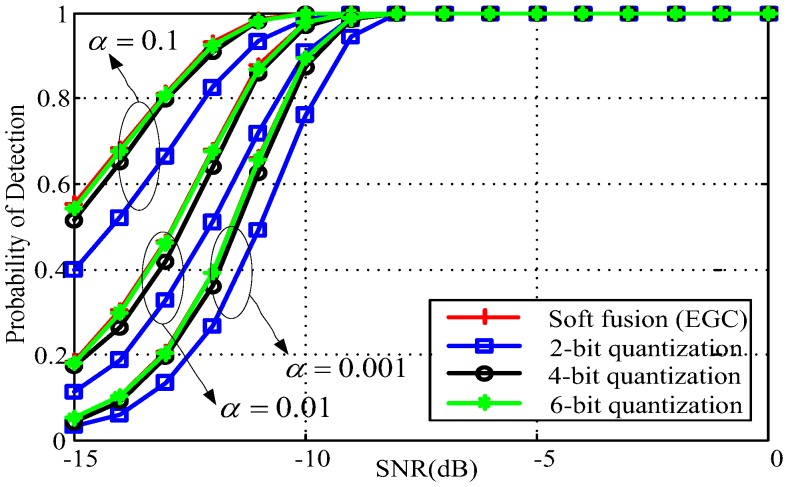
Probability of detection versus signal-to-noise ratio (SNR) for various number of quantization bits and various values for α.

We also verify the performance of the proposed quantization-based multibit soft fusion scheme with different values of quantization bits *q* and different values for global probability of false alarm α, where we opt *q* = 2, 4, 6, and α = 0.1, 0.01, 0.001. From Figure 6, it can be observed that 4-bit and 6-bit quantization-based soft fusion rules achieve comparable detection performance for each value of α, but outperform those achieved by 2-bit quantization-based soft fusion. Furthermore, for a given SNR that is less than −8 dB, the probability of detection is improved by increasing the probability of false alarm and the number of quantization bits.

### 4.2. Detection Probability versus the Number of Samples

**Figure 7:**
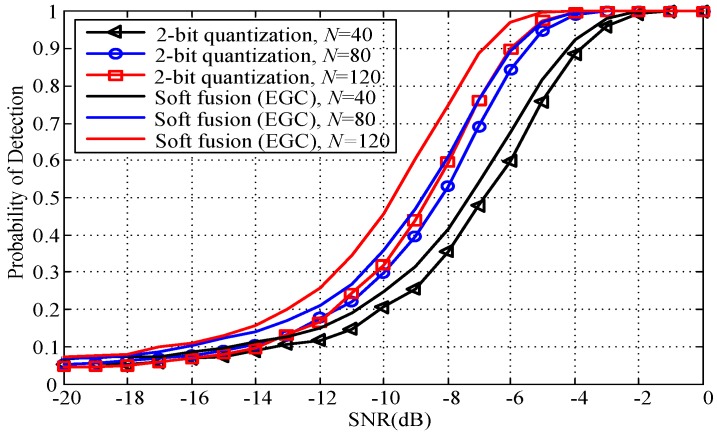
Probability of detection versus SNR for different values of *N* (*M* = 4, *Q_f_* = 0.05).

In general, increasing the samples of the received signal improves the spectrum sensing detector’s performance. Unfortunately, increasing the sensing interval will increase delay in spectrum sensing and lead to a loss in opportunities for spectrum access. Figure 7 shows the detection probability versus the SNR under various numbers of samples for the proposed 2-bit quantization fusion rule. Figure 7 shows that the detection probability is improved when the number of samples increases from 40 to 80 and from 80 to 120. It is also clear that this is true for the soft (EGC) fusion rule. For instance, given SNR = −6 dB, *M* = 4, and *Q_f_* = 0.05, if the required detection probability is 0.8, we can select an *N* of at least 80.

**Figure 8:**
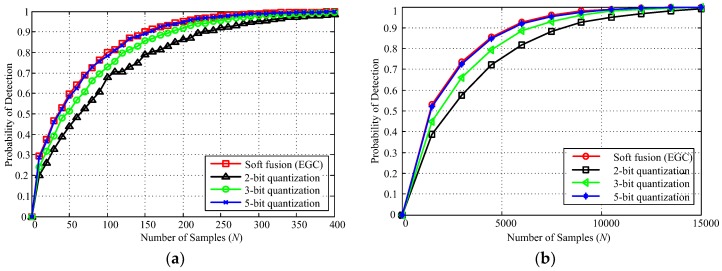
The probability of detection versus the number of samples (*Q_f_* = 0.05, *M* = 4) (**a**) SNR = −8 dB (**b**) SNR = −16 dB.

Figure 8a,b illustrate the detection probability with the number of samples varying from 0 to 400 for an SNR of −8 dB and from 0 to 15,000 for an SNR of −16 dB, respectively. Simulation results in Figure 8 shows that the 5-bit quantization-based soft fusion rule achieves a performance comparable to the soft (EGC) fusion rule with various numbers of samples. For a fixed *N*, increasing quantization bits *q* of each cooperative SU can enhance the probability of detection. We also found that the performance gain of the proposed fusion rule is relatively higher between quantization bits *q* = 2 and 3 than the quantization bits *q* = 3 and 5, respectively. For example, if the required detection probability is more than 0.9, based on Figure 8, we can determine the required number of samples to meet the desired detection probability under SNR = −8 and −16 dB, respectively. In order to meet the detection probability *Q_d_* = 0.9, for −8 dB, the proposed quantization fusion rule requires the number of samples to be approximately 230, 200, and 150 with quantization bits *q* = 2, 3, and 5, respectively. Similarly, for −16 dB, quantization bits *q* = 2, 3, and 5 require the number of samples to be about 7600, 6000, and 5200, respectively. Therefore, at low SNR conditions, more bits and a large number of samples are required to further improve detection performance.

### 4.3. Detection Probability versus the Number of Cooperative SUs

**Figure 9:**
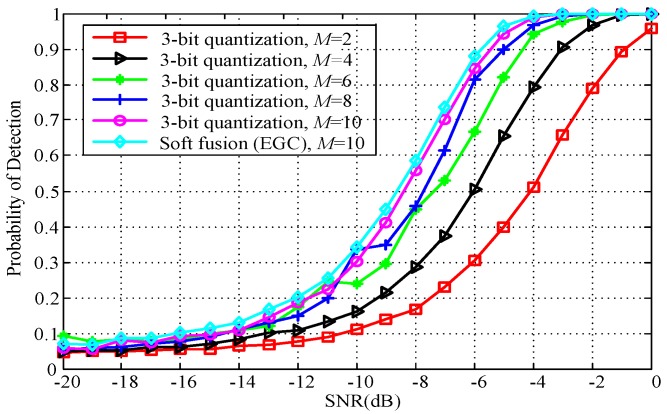
Probability of detection versus SNR for different number of cooperative SUs (*Q_f_* = 0.05, *N* = 30).

To further evaluate the detection performance of the proposed fusion rule, Figure 9 shows the probability of detection versus the SNR for various *M* with 3-bit quantization. It is shown that the detection probability increases by increasing the number of cooperative SUs. It is also clear that, when SNR is greater than −10 dB, the probability of the detection gain is high between *M* = 2, *M* = 4, and *M* = 6 cases of the proposed fusion rule. However, the detection probability gain is relatively low between the *M* = 8 and *M* = 10 cases of the proposed fusion rule. For instance, if we require a detection probability *Q_d_* of 0.8 at an SNR of −6 dB, the required number of cooperative SUs is no less than 8 for 3-bit quantization.

**Figure 10:**
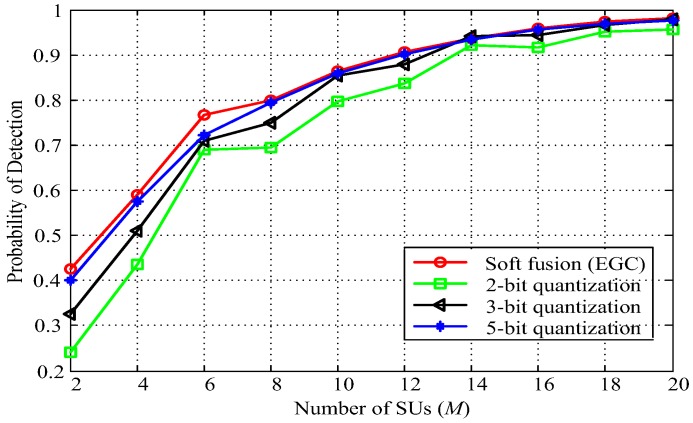
Probability of detection versus number of cooperative SUs (*Q_f_* = 0.1, *N* = 300, SNR = −12 dB).

Figure 10 shows the detection probability in terms of the number of cooperative SUs varying from 2 to 10, where *q* = 2, 3, and 5, and SNR = −12 dB. It is shown that the detection performance is improved by increasing the number of quantization bits and cooperative SUs. Under certain *q* and SNR values, we can use the obtained curve to determine the number of cooperative SUs for a required sensing performance. For instance, to meet the required detection probability *Q_d_* = 0.9 at SNR = −12 dB, the required cooperative SUs *M* is 14, 13, and 12 in cases of the proposed quantization fusion rule where quantization bits *q* = 2, 3, and 5, respectively. The results presented in Figure 10 also show that the performance gain between 2-bit, 3-bit, and 5-bit cases of the proposed quantization-based multibit data soft fusion rule can be negligible when the number of cooperative SUs is more than 14. It is also clear that we can increase the number of cooperative SUs *M* or quantization bits *q* to meet the required detection probability.

**Figure 11:**
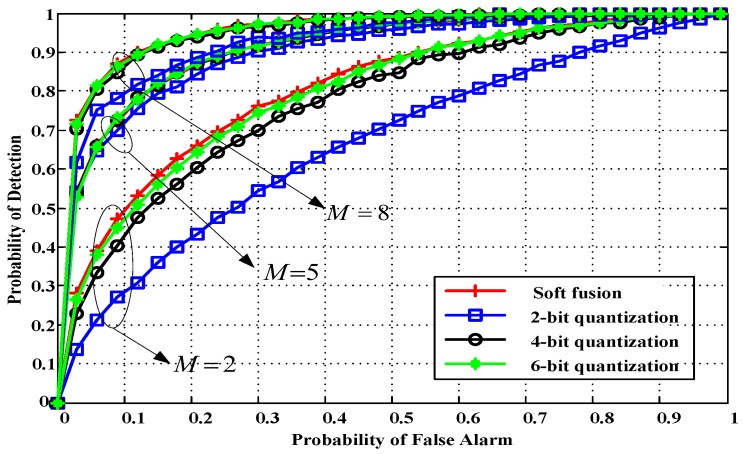
ROC curves for different number of cooperative SUs (SNR = −12 dB, *N* = 400).

The sensing performance with a different number of cooperative SUs, *M*, is shown in Figure 11 for quantization bits *q* = 2, 4, and 6. When the number of cooperative SUs is relatively high, the performance gain improves slightly by increasing the quantization bits *q*. Hence, diversity information among different cooperative SUs further improves detection performance.

## 5. Conclusions

In this paper, we propose a simple quantization-based multibit data fusion scheme for CSS in CRNs. Compared with the hard decision rule (which reports one-bit data to the FC) and the soft fusion rule (which reports observed original statistics to the FC), under our scheme, each SU only reports *q*-bits quantized data to the FC. From the perspective of global false alarm probability at the FC, we determined the center quantization threshold according to the Neyman–Pearson criterion. Meantime, we establish the “3σ” rule to design the quantization interval. The closed-form expressions of quantization levels and quantization thresholds were derived. We also investigated the impact of the number of quantization bits, the SNR, the number of samples, and the number of cooperative SUs on the sensing performance. Extensive numerical simulation results were used to evaluate the proposed quantization fusion rule in comparison with hard decision, soft fusion, SLMC fusion, and semi-soft fusion rules. Simulation results demonstrated that the proposed fusion rule achieved better performance with low computational complexity, and a desirable tradeoff between the detection performance and the control channel’s communication overhead. In our future work, spectrum sensing data falsification, also known as byzantine attack [28], will be involved in the proposed quantization-based multibit data fusion scheme.
